# Evaluation of behavioral variance/covariance explained by the neuroimaging data through a pattern‐based regression

**DOI:** 10.1002/hbm.26601

**Published:** 2024-03-15

**Authors:** Di Chen, Tianye Jia, Wei Cheng, Sylvane Desrivières, Andreas Heinz, Gunter Schumann, Jianfeng Feng

**Affiliations:** ^1^ Institute of Science and Technology for Brain‐Inspired Intelligence Fudan University Shanghai China; ^2^ Key Laboratory of Computational Neuroscience and Brain‐Inspired Intelligence (Fudan University) Ministry of Education Shanghai China; ^3^ Institute of Psychiatry, Psychology & Neuroscience SGDP Centre, King's College London London UK; ^4^ Department of Psychiatry and Psychotherapy CCM Charité—Universitätsmedizin Berlin, Corporate Member of Freie Universität Berlin, Humboldt‐Universität zu Berlin, and Berlin Institute of Health Berlin Germany; ^5^ Department of Computer Science University of Warwick Coventry UK

**Keywords:** brain‐associated variance, neuroimaging, neuroimaging correlation, statistical association

## Abstract

Neuroimaging data have been widely used to understand the neural bases of human behaviors. However, most studies were either based on a few predefined regions of interest or only able to reveal limited vital regions, hence not providing an overarching description of the relationship between neuroimaging and behaviors. Here, we proposed a voxel‐based pattern regression that not only could investigate the overall brain‐associated variance (BAV) for a given behavioral measure but could also evaluate the shared neural bases between different behaviors across multiple neuroimaging data. The proposed method demonstrated consistently high reliability and accuracy through comprehensive simulations. We further implemented this approach on real data of adolescents (IMAGEN project, *n* = 2089) and adults (HCP project, *n* = 808) to investigate brain‐based variances of multiple behavioral measures, for instance, cognitive behaviors, substance use, and psychiatric disorders. Notably, intelligence‐related scores showed similar high BAVs with the gray matter volume across both datasets. Further, our approach allows us to reveal the latent brain‐based correlation across multiple behavioral measures, which are challenging to obtain otherwise. For instance, we observed a shared brain architecture underlying depression and externalizing problems in adolescents, while the symptom comorbidity may only emerge later in adults. Overall, our approach will provide an important statistical tool for understanding human behaviors using neuroimaging data.

## Graphical abstract

We proposed an MRI‐based strategy that could investigate the overall brain‐associated variance (BAV) for a given behavioral measure and evaluate the shared neural bases (i.e., neuroimaging correlation) between different traits. Using large adolescent and adult neuroimaging datasets, we estimated different levels of BAVs and latent brain‐based correlations across multiple phenotypes.

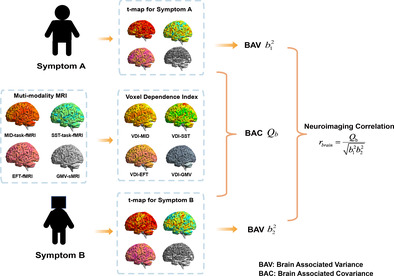

## 1 INTRODUCTION

Understanding the complex relationship between neuroimaging and human behaviors is a fundamental goal of neuroscience. For this purpose, functional and structural MRI (fMRI and sMRI) as non‐invasive neuroimaging techniques with excellent spatial resolution have been widely used to investigate potential neural risk factors (Bulik‐Sullivan, Loh, et al., 2015; Ing et al., 2019; Jia et al., 2020). While both voxel‐ and cluster‐level association analyses (Cheng et al., 2015; Eklund et al., 2016; Ge et al., 2012; Gong et al., 2018) have been widely applied to search for brain‐wide neural bases of human behaviors, current approaches only end up with a few localized brain regions after stringent corrections for multiple testing, hence lacking a whole‐brain overview (Eklund et al., 2016; Gong et al., 2018). For complex behavioral measures, the relevant brain regions may distribute throughout the whole brain (Zhao et al., 2021), and each contributes a small fraction of the overall effect. In such a circumstance, it is a pressing matter to properly estimate the overall brain‐associated variance (BAV) using a whole‐brain approach.

While a few studies have attempted to measure and compare BAV by the neuroimaging data through the intricated brain pattern (Sabuncu et al., 2016; Zhao et al., 2021). As far as we know, there have not previously been reported using neuroimaging data to measure the whole‐brain pattern‐based correlation through neuroimaging between different behavioral measures.

On the other hand, the linkage disequilibrium (LD) score regression (Bulik‐Sullivan, Loh, et al., 2015) has become a widely applied approach in genetic studies to estimate both the systematic confounding effects (such as the population stratification) and SNP heritabilities (i.e., the variance explained by SNPs altogether) of complex behavioral measures based on the summary statistics of genome‐wide association studies (GWAS) (Bulik‐Sullivan, Loh, et al., 2015; Sullivan & Geschwind, 2019). As an extension of the LD‐score regression, the genetic correlation (Bulik‐Sullivan, Finucane, et al., 2015) was then proposed to evaluate the shared genetic constructs between two behavioral measures out of their genetic signals from GWAS and has been applied to investigate genetic correlations among multiple psychiatric disorders (Anttila et al., 2018; Sullivan & Geschwind, 2019).

Inspired by the LD‐score regression (Bulik‐Sullivan, Loh, et al., 2015) and genetic correlation (Bulik‐Sullivan, Finucane, et al., 2015), we proposed a strategy to investigate the variance and covariances of behavioral measures that could be explained by the brain MRI based on a voxel‐based brain‐wide pattern (i.e., voxel dependence index), a measurement of either co‐activations in task‐fMRI or similarities in structure‐MRI. To assess the performance of the model, we conducted a series of simulations with this pattern‐based model and evaluated the accuracy and precision of the estimations. We implemented this method on large real data of adolescents (*n* = 2089) from the IMAGEN project (Schumann et al., 2010) and adults (*n* = 808) from the human connectome project (HCP) (Van Essen et al., 2012) to investigate multiple MRI‐based variances or covariances of cognitive and behavioral measures. This generally applicable approach thus provided a new instrument to understand the global picture of how brain‐wide neuroimaging information could contribute to behavioral measures.

## 2 MATERIALS AND METHODS

### 2.1 Voxel dependence index

Analog to the LD‐score regression (Bulik‐Sullivan, Loh, et al., 2015) and genetic correlation (Bulik‐Sullivan, Finucane, et al., 2015), we developed the voxel dependence index (VDI) pattern regression models to estimate the behavioral variance and covariance that could be explained by the neuroimaging data. Specifically, the VDI of a given voxel was calculated as a sum of task‐fMRI's co‐activation or structure‐MRI's similarity with other voxels across the brain. We defined the VDI of a given voxel *i* as:

$$V_{i}=\sum_{k\neq i}r_{\mathrm{ik}}^{2}$$

where *r*_ik_ is the *Pearson* correlation between voxel *i* and *k* for either task‐fMRI activation or gray matter volume (GMV). Therefore, a voxel with higher VDI would have higher similarities with other voxels and hence are more likely to capture information represented by other voxels across the brain.

### 2.2 VDI pattern regression model

#### 2.2.1 Brain associated variance

Analog to the LD‐score regression (Bulik‐Sullivan, Loh, et al., 2015), the expected *t* ^*2*^ statistic of voxel *i* is:

$$E\left[t_{i}^{2}|V_{i}\right]=\frac{{\mathrm{Nb}}^{2}}{M}V_{i}+c$$

The expected *t* ^*2*^ is proportional to BAV (i.e., *b* ^*2*^), subject to some constant numbers (i.e., *M*, *N*, and *V*_*i*_). The *N* is the sample size, *M* is the number of voxels, and *V*_*i*_ is the voxel dependence index (VDI) of voxel *i*, that is, $V_{i}=\sum_{k\neq i}r_{\mathrm{ik}}^{2}$. The *b* ^*2*^ is the BAV, analog to the genetic heritability. The intercept *c* can be split into two parts: *c* = *Na* + 1, where *a* is the contribution of confounding biases, and *N* is the number of individuals. Hence, a significant deviation of *c* from 1 would indicate the existence of systematic biases, such as population stratification (Bulik‐Sullivan, Loh, et al., 2015). Analog to the genetic linkage disequilibrium structure, we joined multiple MRI paradigms and modalities (i.e., the brain activations from different functional MRI paradigms, such as the monetary incentive delay task [MID], the stop signal task [SST] and the emotional face task [EFT], and grey matter volume [GMV] derived from the structural MRI) in each individual as MRI_combined_ = [MID, SST, EFT, GMV]. In a similar manner, the combined VDI was established as a joint of VDIs calculated for each of the MID, SST, EFT, and GMV.

The BAV (i.e., *b* ^*2*^) could then be calculated once the regression coefficient of the above formula, that is, $\beta =\frac{{\mathrm{Nb}}^{2}}{M}$ was estimated; also see Figure S1 for the flowchart. Particularly, we implemented a two‐stage linear regression to remove confounding factors (i.e., cryptic relatedness and heteroskedasticity across voxels), which were not considered in a standard generalized linear model (GLM), to improve the precision of BAV estimation. In the first stage, we used the constrained‐intercept linear regression, where the intercept was set to one, to estimate the conditional variance. We then applied the weighted linear regression to estimate the β. Following suggestions from LD‐score regression (Bulik‐Sullivan, Loh, et al., 2015), the regression model was weighted by the reciprocal of the conditional variance function, as estimated in the first step to account for heteroskedasticity (i.e., the *t* ^*2*^ statistics of variants with high VDI normally have higher variance than variants' *t* ^*2*^ statistics with low VDI), multiplying by the reciprocal of the VDI, that is, accounting for the dependence among voxels.

#### 2.2.2 Brain associated covariance

Analog to the VDI pattern regression, two linear regression steps were implemented to get the covariance (i.e., *Q*_*b*_) (Bulik‐Sullivan, Finucane, et al., 2015) by the imaging data as follows:

$$E\left[t_{1i}t_{2i}|V_{i}\right]=\frac{\sqrt{N_{1}N_{2}}Q_{b}}{M}V_{i}+\frac{{\mathrm{QN}}_{s}}{\sqrt{N_{1}N_{2}}},$$

where *t*_ji_ denotes the *t* value for voxel *i* and symptom *j*, *N*_*j*_ is the sample size for symptom *j*, *Q*_*b*_ is the neuroimage covariance, *V*_*i*_ is the VDI of the voxel *i*, *N*_*s*_ is the number of individuals included in both studies (i.e., the size of overlapping sample), and *Q* is the phenotypic correlation among the *N*_*s*_ overlapping samples.

Similar to the VDI pattern regression, a two‐step linear regression was used to estimate the neuroimage covariance (i.e., the brain associated covariance [BAC] *Q*_*b*_) with the imaging data. In the first regression step, we employed a constrained‐intercept linear regression, with the intercept constrained by the overlapping sample size and phenotypic correlation acquired in advance, that is, $\frac{{\mathrm{QN}}_{s}}{\sqrt{N_{1}N_{2}}}$. In the second step, we employed the weighted linear regression to estimate the BAC (i.e., *Q*_*b*_). Notably, due to the highly correlated voxel‐level *t* statistics, we implemented the reciprocal of the VDI as a weight to correct for the voxel dependence. In addition, we applied the reciprocal of the conditional variance estimated from the first step as a second weight (multiplied by the first one) to reduce the over‐representation and inflated covariance of voxels with high VDI, that is, accounting for heteroskedasticity. The above procedure is summarized in Figure S2.

#### 2.2.3 Neuroimaging correlation

**FIGURE 1:**
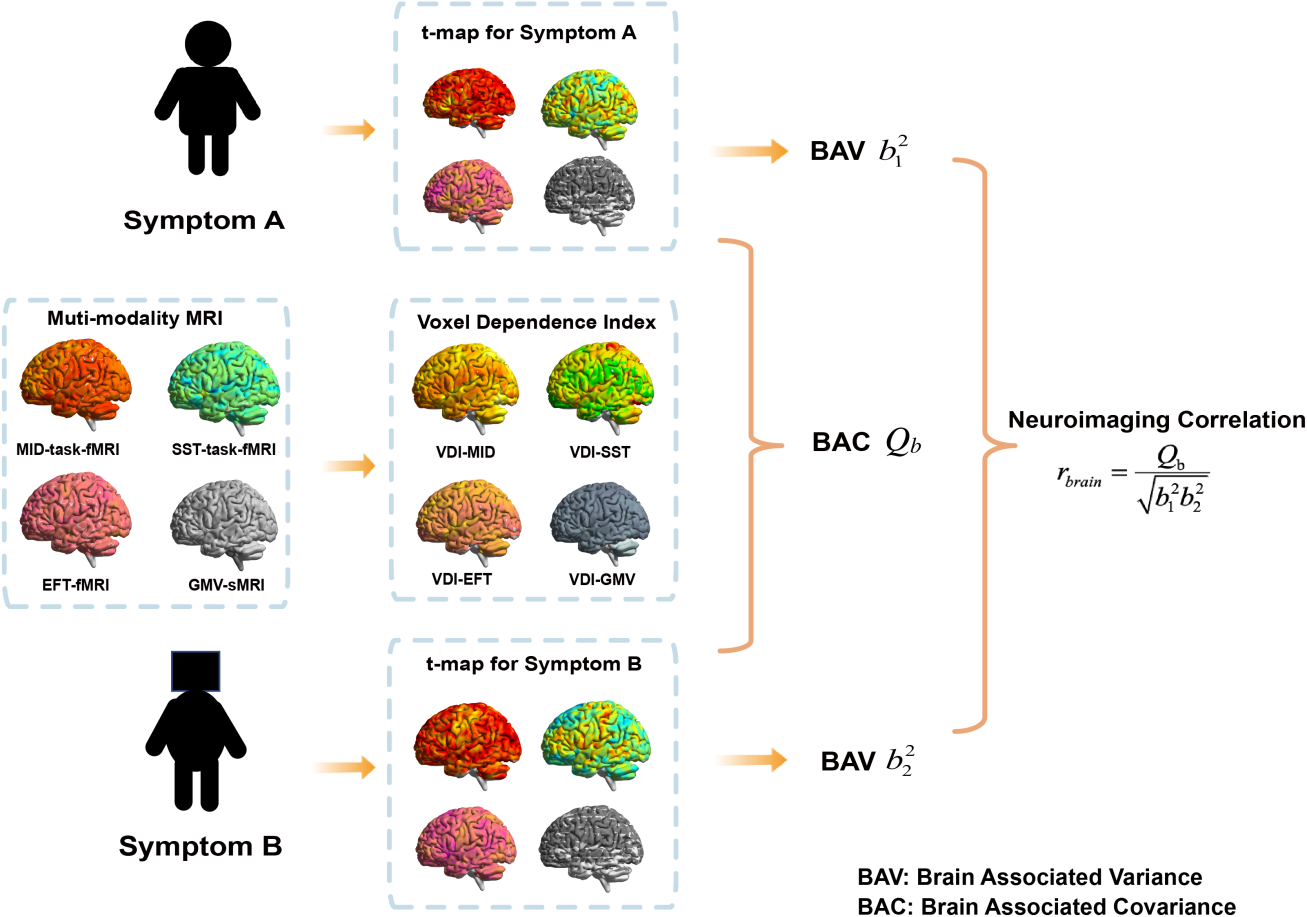
Method overview. Brain‐associated variance (BAV) represents brain‐associated variance; brain associated covariance (BAC) represents brain‐associated covariance; *b*_1_^2^ denotes the BAV of symptom A; *b*_2_^2^ denotes the BAV of symptom B.

The brain‐based correlation *r*_brain_ is defined as follows:

$$r_{\text{brain}}=\frac{Q_{b}}{\sqrt{b_{1}^{2}b_{2}^{2}}},$$

where *b*_*i*_^*2*^ denotes the BAV from symptom *i*, and *Q*_*b*_ is the BAC. The *r*_brain_ evaluates the level of shared brain architecture underlying the paired behaviors. The overview of the method can be found in Figure 1.

### 2.3 Participants

The adolescents (*n* = 2089, Table 1) at the age of 14 from the population‐based IMAGEN project, approved by local research ethics committees (Schumann et al., 2010), were included in the present study. Following the suggestions from previously published papers (Anttila et al., 2018; Jia et al., 2020), we selected 19 behavioral measures from the IMAGEN database, including items covering symptoms of attentional deficit/hyperactivity disorder (ADHD), conduct disorder (CD), anxiety, depression, alcohol, smoking, been bully, bully, exploratory, impulsiveness, extravagance, disorderliness, novelty seeking, performance intelligence quotient (PIQ), and verbal intelligence quotient (VIQ) from the standard questionnaire of strength and difficulties questionnaire (SDQ) (Goodman, 2001), development and well‐being assessment (DAWBA) (Goodman et al., 2000), European school survey project on alcohol and drugs (ESPAD; http://espad.org/). We used a summary score of symptoms to describe the mental disorder (e.g., ADHD symptom) and a summary score for other behaviors (e.g., “Bullying others” for Bully; “being bullied by others” for Been Bully); see Table S1 for details.

**TABLE 1:** Demographic characteristics.

	IMAGEN study	HCP study
Sample size	*n* = 2089	*n* = 808
Age: mean (SD)	14.40 (0.40)	29.08 (3.58)
Sex (male/female)	1023/1065	372/436
Education	/	15.12 (1.69)^a^
Handedness	1862/227 (right/left)	67.59 (42.28)^b^
PIQ^c^: mean (SD)	106.29 (14.21); *n* = 2011	/
VIQ^c^: mean (SD)	79.91 (12.85); *n* = 2011	/
BMI	/	26.3 (4.94); *n* = 807
Household income	/	5.41 (2.02)^d^; *n* = 803

^a^ Years of education completed: <11 = 11; 12; 13; 14; 15; 16; 17+ = 17.

^b^ Handedness of participant from −100 to 100. Negative numbers indicate that a subject is more left‐handed than right‐handed, while positive numbers indicate that a subject is more right‐handed than left‐handed.

^c^ The details of performance IQ and verbal IQ can be found in Table S1.

^d^ Total household income: <$10,000 = 1, 10 K–19,999 = 2, 20 K–29,999 = 3, 30 K–39,999 = 4, 40 K–49,999 = 5, 50 K–74,999 = 6, 75 K–99,999 = 7, ≥100,000 = 8.

The brain network is defined by using the MID‐fMRI to measure reward processing (Knutson et al., 2001), SST‐fMRI to assess motor inhibition (Bari & Robbins, 2013), EFT‐fMRI to examine social–emotional processing (Grosbras & Paus, 2006), and GMV sMRI. In task‐fMRI, we analyzed contrasts that are relevant to the behavior and eliciting the largest BOLD‐difference, namely the “large‐win vs. no‐win” contrast during the reward anticipation phase in the MID, the “successful stop” in the SST, and the “angry face vs. control” contrast in the EFT. For the fMRI signals, the beta values, that is, the activations, derived from the general linear model with the corresponding contrasts were employed in the present study. The details about the MRI data acquisition and standard preprocessing can be found in the previous papers (Jia et al., 2020; Schumann et al., 2010).

**FIGURE 2:**
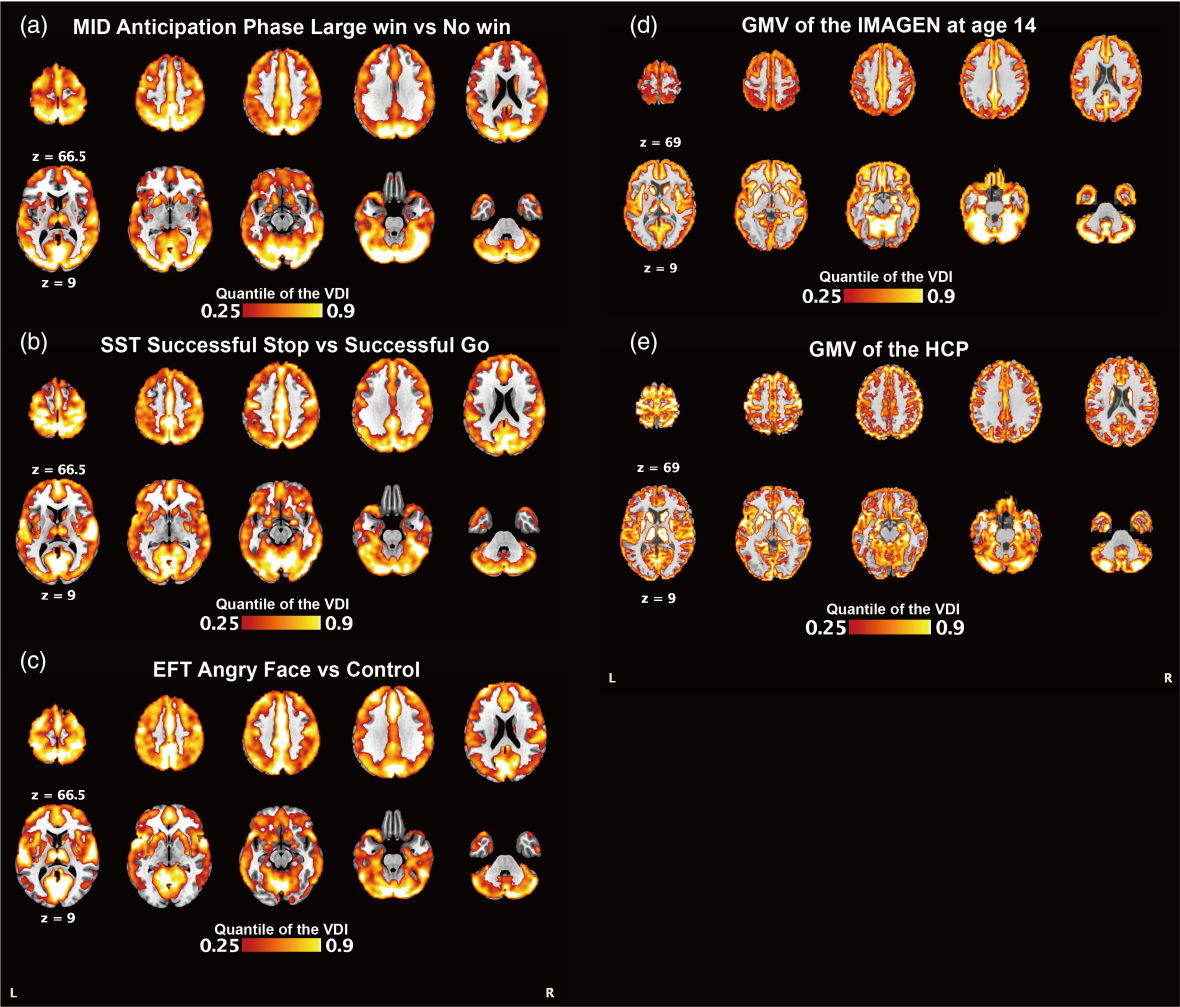
Voxel dependence index (VDI) patterns from the IMAGEN study. The VDI of a given voxel was calculated as a sum of task‐fMRI's co‐activation or structure‐MRI's similarity with other voxels across the brain.

Outliers (*n* = 135 for the MID; *n* = 132 outliers for the SST; *n* = 109 outliers for the EFT; *n* = 112 outliers for GMV) were identified if showing negative correlations with most of the rest of the individuals in terms of the whole brain patterns and hence removed. After imaging quality control, 1820 adolescents for MID‐fMRI, 1890 adolescents for SST‐fMRI, 1889 adolescents for EFT‐fMRI, and 1979 adolescents for GMV‐sMRI were employed in the present study. To maintain consistency with functional imaging, after the completion of image preprocessing, we resampled all structural images to a 3 mm standard level by AFNI (Cox, 1996) for all datasets. After this approach, we identified the combined MRI paradigms and modalities (i.e., MID, SST, EFT, and GMV) consisting of over 210,000 voxels. Task fMRI of the MID task, SST, and emotional faces task, and sMRI of GMV were used to calculate the corresponding VDIs for the IMAGEN study (Figure 2). The high similarities between fMRI VDI maps are in line with our previous observations that different task‐fMRIs demonstrated similar activations (Jia et al., 2020). The moderate similarity between structural MRI and functional fMRI is likely due to their differentiated underlying biological processing, where fMRI measures the BOLD signal that is closely related to the dynamic neuron activity, and structural MRI measures the gray matter volume, a static measurement of the neuronal cell density. Finally, the moderate similarity between structural MRIs is likely because of age differences, where IMAGEN participants were 14 years old, and the HCP dataset included individuals with an average age of 29. The gender, handedness, and research sites were included as control variables for the *t* value in simulation settings and real data.

The adults (*n* = 808, Table 1) from the HCP study (Van Essen et al., 2012) were investigated for the BAVs of the 10 behavioral measures, including psychiatric disorders, cognition behaviors, and substance use; see Table S2 for details. Gender and age were included as control variables for the *t*‐value in the HCP analysis. To minimize ethnic heterogeneity and keep in line with the IMAGEN study, only whites from the HCP were included in the following analyses. For the GMV in the HCP, the pre‐processing was also implemented by the standard pipeline, which is in line with the IMAGEN.

### 2.4 Simulations

To evaluate the performance of our proposed model for the estimation of BAV, we conducted various simulations using the VDI pattern regression model. The workflow for generating the phenotype in each simulation is as follows: (i) we randomly select a voxel in the real MID‐task fMRI data; (ii) we use the function “zscore” from Matlab to standardize the signal vector (with length *n* = sample size) of the selected voxel, that is, normalizing the signal to have mean zero and a standard deviation of one; (iii) then, we randomly generate noise signal with Matlab function “normrnd” with mean zero and a set of predefined standard deviation; (iv) finally, we sum the standardized *Z*‐score and noise to generate the simulated phenotype as follows: simulated phenotype = real‐data signal at the selected voxel (*Z*‐scored) + noise. We conducted simulations 100 times, and each time, a simulated phenotype was generated from a randomly selected single voxel (i.e., the 100 times simulations corresponding to 100 randomly selected voxels). The real MID‐fMRI data was employed in the BAV simulation analyses. The expected BAV (Table S3) were set at 0%, 5%, 10%, 20%, and 50%.

We then performed a series of neuroimaging correlation simulations for overlap‐sample simulation to evaluate the model's precision and robustness. We first generated two phenotypes based on the MID fMRI data for each of 1820 individuals, where both real‐data signal and noise follow the same normal distribution with *mean* = 0, *std* = 1. Second, we generated one phenotype from MID and the other from SST (*n*_MID_ = 1820, *n*_SST_ = 1890, *n*_intersect_ = 1683). In addition, we performed a series of simulations that generated two phenotypes combining the MID task and SST task, but with different contributions (Table S4), that is, by varied variance ratios. In each simulation setting, the average neuroimaging correlation *r*_brain_ was estimated after 100 simulations. In rare occasions, negative BAVs (very close to 0) could be observed and were hence excluded in the following calculation for neuroimaging correlations. A similar process was performed for sample‐independent simulation in which one phenotype came from the male while the other came from the female to guarantee the sample's independence. The similarity (i.e., root mean squared error [RMSE]) between the observed and expected BAV or neuroimaging correlation was employed to evaluate the simulation's performance.

### 2.5 Statistic inference

We recommended a “quick” *p*‐value procedure using a pre‐calculated 95% confidence interval (CI) of BAV for each type of MRI through simulations, where we generated a random phenotype following the normal distribution with “mean = 0, std = 1” for 1000 times and then established a null distribution of BAV for the random phenotype. This procedure could provide an approximate *p*‐value that does not require the knowledge of the exact phenotypic distribution and hence could be used as a quick scanning for potentially significant findings. To assess the accuracy of the quick *p*‐value, we also compared the quick *p*‐values with 1000 times permutation *p*‐values on real GMV‐based data of the IMAGEN project. The consistent result between the two procedures indicates the high precision and reliability of the quick *p* value procedure; see Table S5 for details.

Nevertheless, to give a precise p‐value for the BAV, we would recommend randomly shuffling the behavioral measures across individuals to get the permutation *p*‐value in the VDI pattern regression.

Similarly, to get a precise *p*‐value of the neuroimaging correlation, 1000 times standard permutation flow (i.e., shuffling the behavioral measures across individuals) was implemented to estimate the significance level (e.g., *p*_Perm_ <.05) of the neuroimaging correlation in the present study.

### 2.6 The applications to real data

The VDI pattern regression was implemented to estimate the BAVs on the selected behavioral measures from the IMAGEN project (Schumann et al., 2010) and the HCP (Van Essen et al., 2012) dataset in the present study. The standard false discovery rate (FDR) correction (Benjamini & Hochberg, 1995) was implemented for multiple comparisons in each MRI type's BAV estimation. In addition, we also implemented the approach to estimate neuroimaging correlations among behavioral measures through the combined MRI data in the IMAGEN study. For clarity of presentation, the eight behavioral measures were restricted to include only the behavioral measure with a significant (i.e., *P*_FDR_ <0.05) BAV value.

## 3 RESULTS

### 3.1 Estimation of BAV through VDI pattern regression using real‐data simulations

**FIGURE 3:**
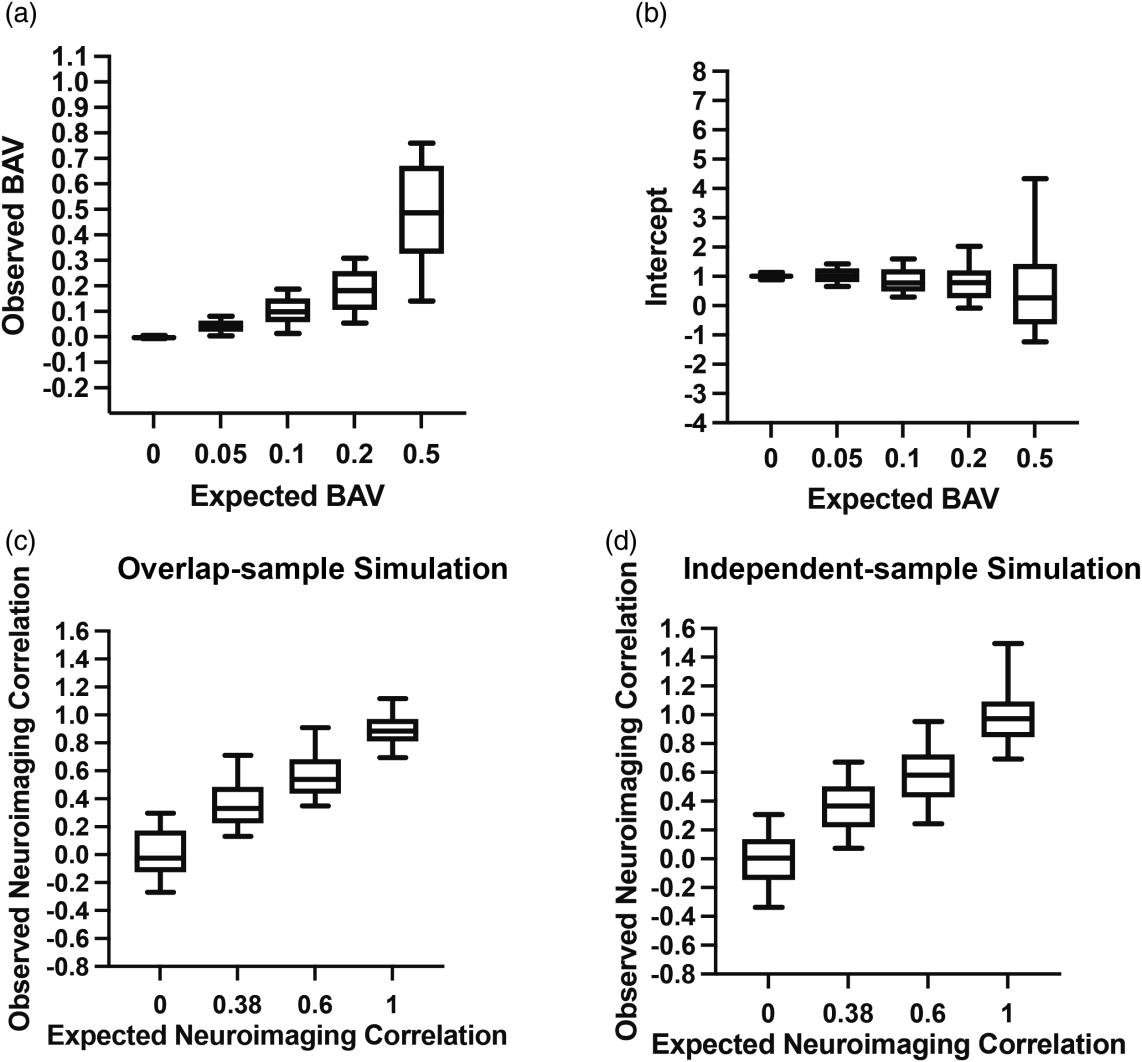
Simulations. (a) The brain‐associated variance (BAV) was estimated by the voxel‐based simulation from 100 simulation replicates for each level of expected BAV; (b) The intercept of the voxel dependence index (VDI) pattern regression model from 100 times simulation replicates for each level of expected BAV; (c) Simulations of neuroimaging correlation for overlap‐sample; (d) Simulations of neuroimaging correlation for independent‐sample.

To assess the reliability of our method, we performed various real‐data simulations that generated hypothetical phenotypes with various scenarios (Table S3), levels of cryptic relatedness, and brain architecture observed in real data, that is, the MID task fMRI from the IMAGEN study. The corresponding BAVs were then estimated with 100 simulation replicates at each of five different presettings (BAV = 0%, 5%, 10%, 20%, 50%). The observed BAVs were highly consistent with their expected values (Figure 3a) at all five simulation settings, that is, mean = −0.001 (std = 0.01), RMSE = 0.006 for 0%, mean = 0.043 (std = 0.03), RMSE = 0.033 for 5%, mean = 0.102 (std = 0.06), RMSE = 0.064 for 10%, mean = 0.182 (std = 0.10), RMSE = 0.098 for 20%, mean = 0.477 (std = 0.25), RMSE = 0.248 for 50%. Further, in all simulation settings, the regression models' mean intercepts were all close to one, indicating that the above simulation results were free from systematic bias, such as unknown population stratification (Figure 3b).

### 3.2 Estimation of neuroimaging correlation using real‐data simulations

We next performed a series of simulations to evaluate the neuroimaging correlation model's performance, as well as its robustness to potential confounders, such as model misspecification. For overlap‐sample simulation, we respectively generated two phenotypes for each of 1820 individuals from MID‐fMRI by drawing effect sizes for approximately 54,000 voxels for each simulation replicate. The mean of observed neuroimaging correlations was close to one (*r*_mean_ = 0.9371, *std* = 0.2647) after 100 times simulation, which is largely due to the strong co‐activation across the brain in the MID task, that is, lacking substructures with independently explained variance (i.e., analog to the linkage disequilibrium structures in the genetic data) across the brain to form a meaningful neuroimaging correlation. Therefore, neuroimaging correlations could only be properly estimated in the presence of multiple MRI paradigms and modalities.

First, we simulated two phenotypes for each individual from the same population (*n*_intersect_ = 1683) using fMRI data of the MID task and the SST, where the contribution ratios of both tasks were based on a series of preset values (Table S4). Based on 100 iterations of simulations, the observed inner‐sample neuroimaging correlations were highly consistent with the expectations (Figure 3c), that is, *r*_mean_ = 0.00 (*std* = 0.27), RMSE = 0.266 for *r* = 0, *r*_mean_ = 0.39 (*std* = 0.26), RMSE = 0.263 for *r* = 0.38, *r*_mean_ = 0.60 (*std* = 0.30), RMSE = 0.295 for *r* = 0.60; *r*_mean_ = 0.91 (*std* = 0.18), RMSE = 0.200 for *r* = 1.00.

Next, we divided the population into two groups by gender (i.e., “male” & “female”; *n*_male_ = 799, *n*_female_ = 884), and in each group, we generated one phenotype using fMRI data of the MID task and the SST, and the contribution ratios of both tasks were based on a series of preset values. Based on 100 iterations of simulations, the observed inter‐sample neuroimaging correlations were again highly consistent with the expectations (Figure 3d), that is, *r*_mean_ = 0.00 (*std* = 0.28), RMSE = 0.281 for *r* = 0, *r*_mean_ = 0.41 (*std* = 0.37), RMSE = 0.364 for *r* = 0.38, *r*_mean_ = 0.62 (*std* = 0.35), RMSE = 0.348 for *r* = 0.60, *r*_mean_ = 1.02 (*std* = 0.33), RMSE = 0.332 for *r* = 1.00.

### 3.3 Application of VDI pattern regression on IMAGEN and HCP data

We first applied VDI pattern regression on multiple behavioral measures to evaluate the BAVs of GMV from 1979 adolescents of the IMAGEN database and 808 adults of the HCP study. Notably, the intercepts of VDI pattern regression were all close to one (Tables S6, S7, S9–S12), hence confirming that the corresponding results were free from systematic bias, such as unknown population stratifications.

**FIGURE 4:**
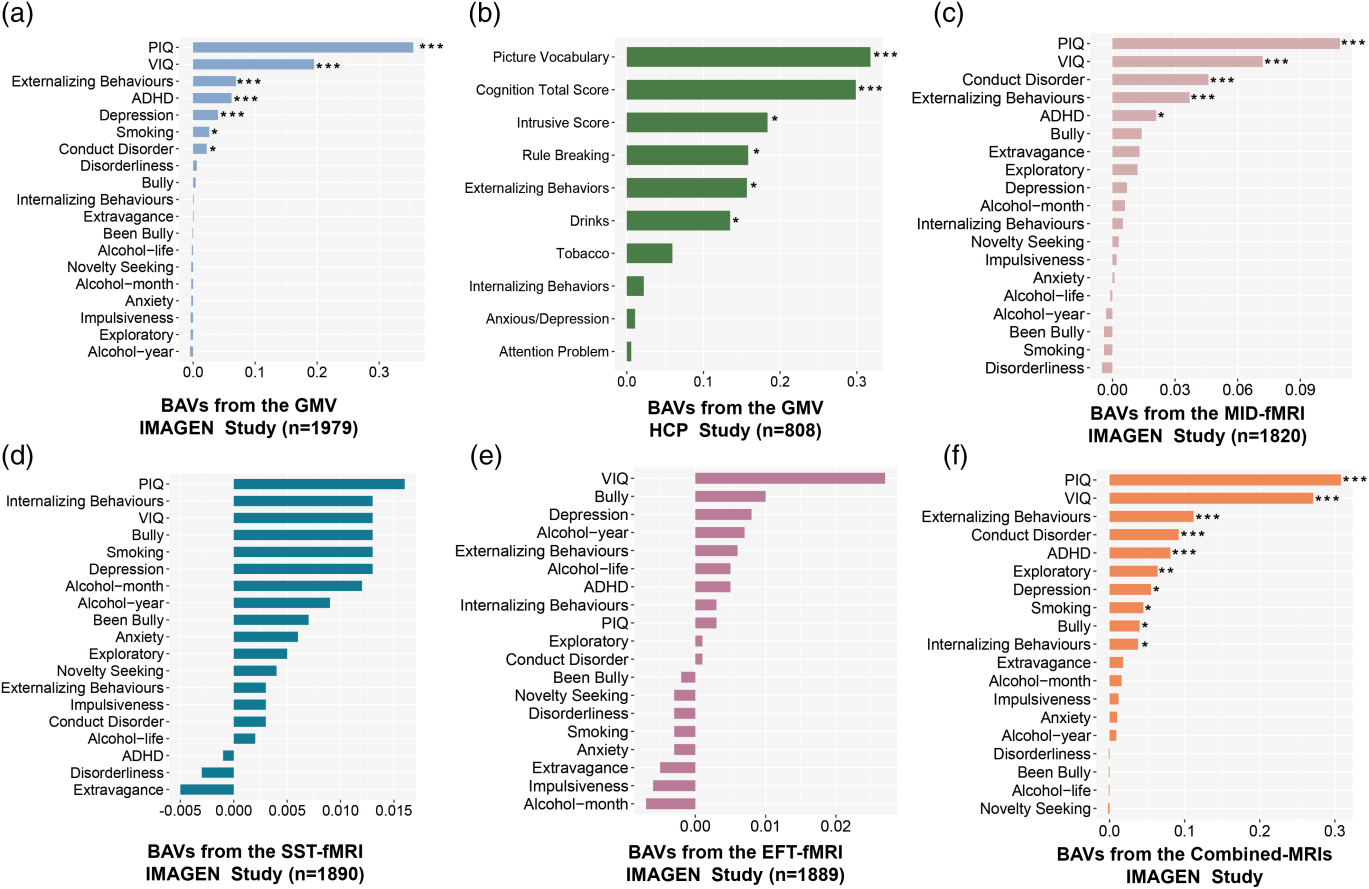
Brain‐associated variances (BAVs) of multi‐type MRI. (a) BAVs of GMV from the IMAGEN study; (b) BAVs of gray matter volume (GMV) from the human connectome project (HCP) study; (c) BAVs of MID‐task fMRI from the IMAGEN study; (d) BAVs of SST‐fMRI from the IMAGEN study; (e) BAVs of EFT‐fMRI from the IMAGEN study; (f) BAVs of Combined‐MRIs from the IMAGEN study. *p_FDR_ <.05; **p_FDR_ <.01; ***p_FDR_ <.001.

Intelligence scores demonstrated high BAVs of GMV in both IMAGEN (BAV = 0.355, *p*_FDR_ <.001 for performance‐IQ; BAV = 0.195, *p*_FDR_ <.001 for verbal‐IQ; Figure 4a, Table S6) and the HCP (BAV = 0.318, *p*_FDR_ <.001 for picture vocabulary; BAV = 0.299, *p*_FDR_ <.001 for cognition total score; Figure 4b, Table S7), which is in line with previous observed consistent genetic heritability for IQ throughout adolescents and adults (Bouchard, 2013).

Significant BAV of GMV was observed for externalizing behaviors in IMAGEN (*BAV* = 0.069, *p*_FDR_ <.001; Figure 4a, Table S6), but not for internalizing behaviors (BAV = 0.001, *p*_FDR_ = .507; Figure 4a, Table S6). For the HCP study, we also observed significant BAVs of GMV for externalizing behavior (*BAV* = 0.157, *p*_FDR_ = .034; Figure 4b, Table S7), but again not for internalizing behavior (*BAV* = 0.022, *p*_FDR_ = .326; Figure 4b, Table S7). These results hence indicated that brain‐wide GMV may mainly contribute to externalizing behaviors through adolescence and adulthood, but less to internalizing behaviors.

We also performed the analyses to use the VDI obtained from the GMV of the IMAGEN dataset (*n* = 1979, age = 14 for IMAGEN‐14; *n* = 1132, age = 19 for IMAGEN‐19) to calculate the BAVs based on the GMV of HCP dataset. We observed significant correlations of BAVs between the HCP and both IMAGEN datasets (Spearman *r* = .95, *p* < .001, RMSE = 0.1551 for the IMAGEN‐14; Spearman *r* = .90, *p* < .001, RMSE = 0.1373 for the IMAGEN‐19; Table S8), indicating the potential comparability of neuroimaging paradigm. The IMAGEN‐19 demonstrated better RMSE (i.e., smaller) with the HCP (age mean = 29.08, std = 3.58) than the IMAGEN‐14, which may be attributed to the relatively smaller age difference.

Further, in the IMAGEN study, with multiple task‐based functional MRI data (i.e., MID, SST, and EFT), we observed significant BAVs for intelligence scores (the MID: BAV = 0.109, *p*_FDR_ <.001 for performance‐IQ, and BAV = 0.072, *p*_FDR_ <.001 for verbal‐IQ; the SST: BAV = 0.016, *p* = .036, *p*_FDR_ = .174 for performance‐IQ; the EFT: BAV = 0.027, *p* = .010, *p*_FDR_ = .190 for verbal‐IQ) and the externalizing total score (BAV = 0.037, *p*_FDR_ <.001 for the MID), but not internalizing behaviors (Figure 4c–e, Tables S9–S11).

Finally, combining all MRI paradigms and modalities (MID, SST, EFT, and GMV) from the IMAGEN study, we observed ten significant BAVs (i.e., *P*_FDR_ <0.05) out of 19 behavioral measures (Figure 4f, Table S12), that is, highest for intelligence scores (BAV = 0.308, *p*_FDR_ <.001 for performance‐IQ; BAV = 0.271, *p*_FDR_ <.001 for verbal‐IQ), moderate for externalizing behaviors (BAV = 0.112, *p*_FDR_ <.001) and lowest in internalizing behaviors (BAV = 0.038, *p*_FDR_ = .046), which were consistent with the above findings from single MRI paradigm. In addition, we now also observed significant BAV in behaviors such as bully (BAV = 0.040, *p*_FDR_ = .038), smoking (BAV = 0.045, *p*_FDR_ = .029), and exploratory behaviors (BAV = 0.064, *p*_FDR_ = .006).

### 3.4 Split‐half analysis

To assess the consistency of the VDI pattern regression, we also conducted a split‐half analysis. Specifically, we randomly divided the IMAGEN individuals into two sub‐sets with equal sample sizes, and separately performed the VDI pattern regression on 23 behavioral measures (Table S13). We then correlated the calculated BAVs of two sub‐sets to determine the reproducibility of the proposed approach.

The split‐half analysis of the whole‐brain VDIs exhibited a high reproducibility across the two half‐split sub‐groups (*r* = .83, *p*_one‐tail_ <.001 for MID, *r* = .77, *p*_one‐tail_ <.001 for SST, *r* = .75, *p*_one‐tail_ <.001 for EFT, *r* = .97, *p*_one‐tail_ <.001 for GMV; Figure S3). Also, the BAVs showed correlation with medium to large effect size (Jacob, 1988) between two sub‐groups based on the whole‐sample VDI (*r* = .62, *p*_one‐tail_ <.001, Figure S4), their respective VDIs (*r* = .46, *p*_one‐tail_ = .014, Figure S4), and even with VDIs estimated only from one of the two sub‐groups (*r* = 0.47, *p*_one‐tail_ = .012, Figure S4). The above results hence indicated a high level of consistency in this approach.

### 3.5 Application of neuroimaging correlation on IMAGEN data

**FIGURE 5:**
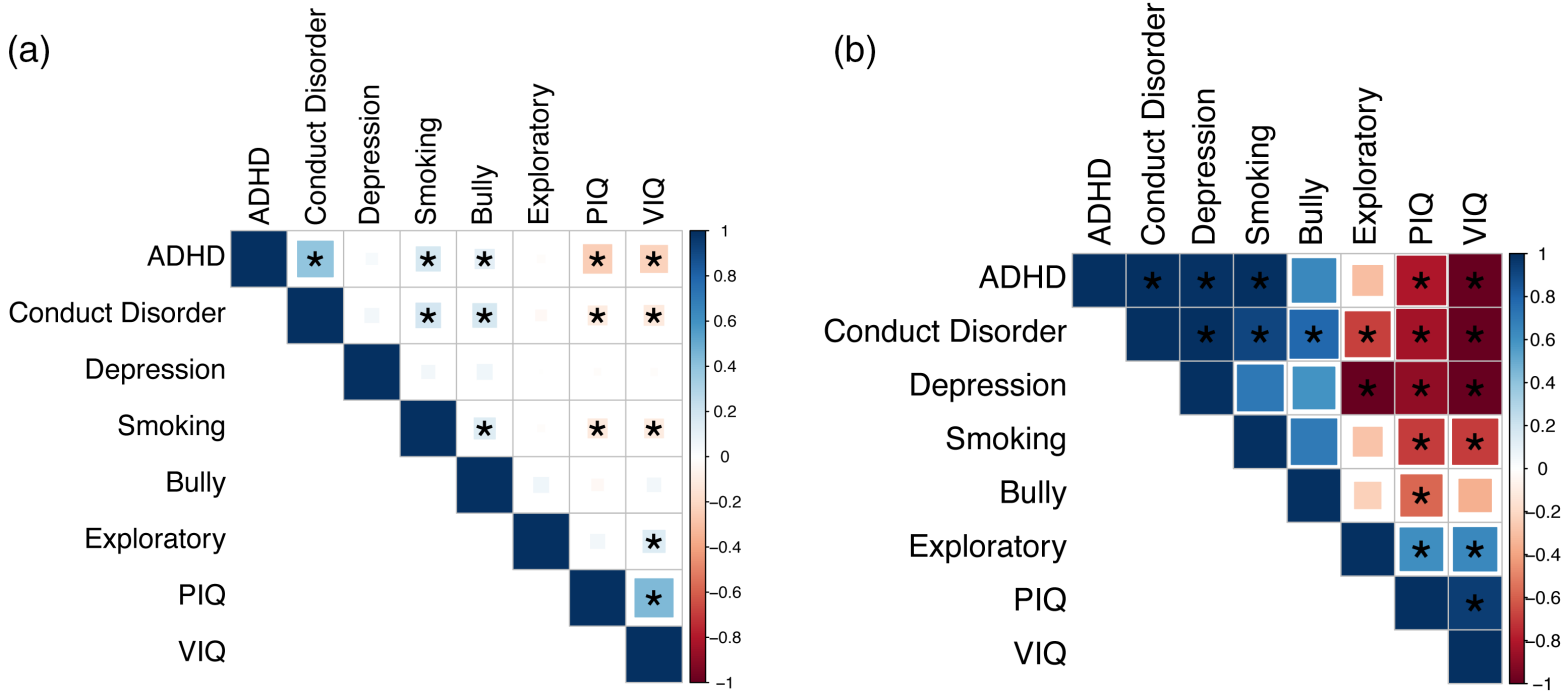
Behavior correlations and neuroimaging correlations. (a) Behavior correlations. (b) Neuroimaging correlations. The absolute *r*_brain_ value was capped at one; see Table S13 for details. * represents the *r* value or *r*_brain_ value is significantly different from zero at significance level 0.05 after *Bonferroni* correction (i.e., *p* < .05/28 = 0.0018).

We implemented the neuroimaging correlation model to estimate brain‐based correlations between eight significant behavioral measures (i.e., ADHD, conduct problems, depressive symptoms, smoking, bully behaviors, exploratory behaviors, performance‐IQ, and verbal‐IQ) based on their BAV shown above, and found that correlations derived from the behavior and brain information were largely aligned with each other (Figure 5a,b; Tables S14–S17). For instance, consistent positive relationships were observed among common behavior problems (i.e., ADHD, conduct disorder, smoking, and bully) during adolescence using both behavior (*r* > .11, *p* < .001) and neuroimaging correlations (*r*_brain_ >.64, *p*_Perm_ <.05), and these behavior measures were further anticorrelated with higher IQ scores in terms of both behavior and neuroimaging correlations.

However, with neuroimaging correlations, we also had several exclusive findings that would have been difficult to obtain otherwise. For instance, we observed almost perfect positive neuroimaging correlations between depressive symptoms and externalizing behaviors (e.g., *r*_brain_ = 0.99, *p*_Perm_ <.001 between ADHD and depressive symptoms; Figure 5b, Tables S16 and S17), which cannot be obtained using behavior information alone (Figure 5a, Tables S14 and S15). Also, exploratory behavior was found with strong positive neuroimaging correlations with both IQ scores (*r*_brain_ = .62, *p*_Perm_ <.001 with performance‐IQ; *r*_brain_ = .64, *p*_Perm_ <.001 with verbal‐IQ; Figure 5b, Tables S16 and S17), as well as strong negative neuroimaging correlation with conduct problems (*r*_brain_ = −.69, *p*_Perm_ = .001; Figure 5b, Tables S16 and S17), which were either much weaker or non‐significant if using behavior information alone (Figure 5a, Tables S14 and S15).

## 4 DISCUSSION

In the present study, we introduced the VDI‐based regression to evaluate the BAV of behavioral measures. The voxel dependence index, which measures the similarity of a particular voxel with others, could potentially be established from independent data so long as the neuroimaging paradigm and age band are comparable. Further, this VDI‐based regression could be applied to a pair of behavioral measures of interest, evaluating to what extent the neuroimaging data could simultaneously explain both behavioral measures, that is, the neuroimaging correlation.

The proposed approach was implemented on 2089 adolescents from the IMAGEN project (Schumann et al., 2010) to explore the BAV of multiple behavioral measures and found that: Externalizing behaviors, that is, ADHD and conduct problems, could be significantly explained by the MID task and GMV, and both unsurprisingly shared a high neuroimaging correlation as expected (Figure 5b); Depressive symptoms (BAV = 0.040, *p*_FDR_ <.001), but not anxiety, showed a significant BAV with the GMV. Such a difference is significant (BAV_diff_ = BAV_depress_−BAV_anxiety_ = 0.0436, *p*_permu_ = .002 based on 1000 times permutation), indicating that the GMV may serve as a biomarker to distinguish the two internalizing brain disorders; The bullier, but not victims, has a small but significant BAV with the MID task, indicating a deficit in reward processing of the adolescents with the bully problem, which agrees with previous social‐reward reports (Guy et al., 2019); Both PIQ and VIQ showed a high total BAV value (>27%), especially with the GMV (i.e., 36% for PIQ and 20% for VIQ), which reconfirmed the well‐known high relevance of GMV for the intelligence quotient (Genon et al., 2022; Hidese et al., 2020; Yoon et al., 2017), but for the first time providing a reliable estimation for the overall explained variance.

We also implemented this approach on 808 adults in the HCP study (Van Essen et al., 2012) to investigate GMV BAVs of psychiatric symptoms, intelligent‐related behaviors, and substance use. Analog to the above IMAGEN results, intelligent‐related behaviors demonstrated the highest BAVs, while externalized symptoms and substance use had moderate BAVs, which is consistent with findings in previous studies (Chen et al., 2022; Genon et al., 2022; Jia et al., 2020; Sun et al., 2023), where significant correlations have been observed between these behavioral measures and brain imaging. Therefore, we have observed consistent BAVs using two different cohorts at different life stages (i.e., adolescents of the IMAGEN and adults of the HCP) and hence reaffirmed the reliability of our new approach.

Further, our approach allows us to investigate neuroimaging correlations using multiple MRI paradigms and modalities. For instance, daily smoking frequency and bully behaviors showed positive neuroimaging correlations with multiple psychiatric illnesses, including ADHD, conduct problems, and depressive symptoms, and is in line with behavior correlations in the present study, as well as from previous findings (Copeland et al., 2013; Gilbody et al., 2019; Quinlan et al., 2020). We also observed positive neuroimaging correlations between exploratory behaviors and IQ scores, all of which further demonstrated negative neuroimaging correlations with both externalizing and internalizing behaviors, again consistent with our behavior correlation, as well as previous findings (Hilger et al., 2020; Keyes et al., 2017). However, it is noteworthy that unlike the high GMV BAVs (≥20%) of IQ scores, exploratory behaviors were hardly explained by GMV (BAV = 0%), suggesting that the shared neural bases (*r*_brain_ >.60) between exploratory behaviors and IQ scores were largely represented by context‐dependent brain activations across different tasks, but not the context ambiguous GMV.

While multiple variable approaches (e.g., canonical correlation analysis CCA, support vector regression, and morphometric analysis [Sabuncu et al., 2016]) could also assess the relationship between behavioral measures and whole brain pattern, the proposed brain‐pattern analysis could additionally adjust for confounding factors such as relatedness between voxels and heteroskedasticity through the weighted linear regression. Also, methods like CCA tend to overestimate the explanatory variance and hence require appropriate adjustment to correct for the overfitting (Jia et al., 2020), while the proposed method is unbiased (Bulik‐Sullivan, Loh, et al., 2015). Further, it is a common issue that there are a higher number of features than the sample size (for instance, in our case), and hence regularizations (e.g., ridge or/and lasso) are required for multiple variable approaches to avoid matrix singularities (i.e., due to insufficient degree of freedom), which, however, will inevitably underestimate the variance explained (Jia et al., 2020). Finally, it is only through the proposed brain‐pattern approach that we could estimate the neuroimaging correlation, which helps to reveal the latent neural relationships shared between different behavioral measures. For instance, unlike behavior‐based correlations (Figure 5a), we observed significant positive neuroimaging correlations between depressive symptoms and externalizing behaviors (including ADHD and CD) (Figure 5b), indicating a shared brain architecture underlying externalizing and internalizing behaviors, although the symptom‐level comorbidity might only emerge at a later time in adults.

The present study is not without limitations. We only included white people from the IMAGEN and HCP cohorts to minimize ethnic heterogeneity. We would need to validate our current approach and findings in different ethnic groups in the future. We acknowledge that as the simulated BAV increases, the precision of the BAV estimation will reduce (i.e., with amplified variance), and the intercept estimation could be biased. These effects become prominent when the expected BAV reaches 50%. Unfortunately, the underlying mechanism is unclear, although the amplified variance might be related to the restricted size and sparsity of the brain network (manifested as the VDI). Nevertheless, the highest BAV was estimated at around 30% for IQs, and therefore, the above issue is likely to have a minimal impact in practice. Further, while our BAV findings of multiple symptoms are in line with previous observations (Chen et al., 2022; Jia et al., 2020), we also acknowledge that additional MRI paradigms and modalities would further enhance the precision of neuroimaging correlation estimations.

## 5 CONCLUSION

In conclusion, we introduced a robust VDI pattern regression to provide an unbiased estimation of BAV of different behavioral measures. Further, using only summary statistics, this method could also reveal the shared neural bases across different mental disorders and cognitive behaviors that might have been difficult to obtain from symptom‐based approaches, hence providing more insights into the neural mechanisms underlying comorbid mental disorders. Finally, we successfully applied our method to two real population‐based databases of adolescents (IMAGEN project, *n* = 2089) and adults (HCP project, *n* = 808). Analog to the LD‐score regression and the genetic correlation, we believe our approach will provide an important advance in the field of neuroimaging studies.

## AUTHOR CONTRIBUTIONS

*Design of study*: T.J., J.F., D.C. *Manuscript writing and editing*: D.C. and T.J. wrote the manuscript; all authors critically reviewed the manuscript. *Data acquisition*: T.J., S.D., A.H., G.S. *Data analysis*: D.C. and T.J.

## CONFLICT OF INTEREST STATEMENT

The authors declare no financial interests or personal relationships which may be considered as potential competing interests.

## Supporting information


**DATA S1:** Supporting Information.

## Data Availability

The IMAGEN and Human Connectome Project are available from dedicated databases at imagen2.cea.fr and www.humanconnectome.org after presenting a request. The code of VDI pattern regression and neuroimaging correlation can be obtained at https://github.com/dichen27/VDI_Regression.
