# Supplementary material for: Evaluation of behavioral variance/covariance explained by the neuroimaging data through a pattern‐based regression
Source: Hum Brain Mapp. 2024 Mar 15;45(4):e26601. doi: 10.1002/hbm.26601 (PMC10941514; doi:10.1002/hbm.26601)
Supplement: Supplementary file 1 — DATA S1: Supporting Information. [file HBM-45-e26601-s001.pdf]

Supplementary material

Supplementary Tables 2

Table S1. Descriptions of behavioral measures from the IMAGEN project 2
Table S2. Descriptions of behavioral measures from the HCP study 4
Table S3. Simulations of BAVs with different scenarios 4
Table S4. Simulations of neuroimaging correlation with different scenarios 4
Table S5. Permutation P-values of the GMV-based BAVs from the IMAGEN 5
Table S6. BAVs of GMV from the IMAGEN project 6
Table S7. BAVs of GMV from the HCP study 6
Table S8. BAVs of HCP Phenotypes with various VDI pattern 7
Table S9. BAVs of MID-fMRI from the IMAGEN project 7
Table S10. BAVs of SST-fMRI from the IMAGEN project 8
Table S11. BAVs of EFT-fMRI from the IMAGEN project 9
Table S12. BAVs of Combined-MRI from the IMAGEN project 10
Table S13. Phenotypes in split-half analysis (age 14) 11
Table S14. R-values of behavior-based correlations 12
Table S15. P-values of behavior-based correlations 12
Table S16. Rbrain-values of neuroimaging correlation 13
Table S17. P-values of neuroimaging correlation 14

Supplementary Figures 15

Figure S1. Flowchart of VDI pattern regression. 15
Figure S2. Flowchart of neuroimaging correlation. 16
Figure S3. Split-half analysis of VDIs. 17
Figure S4. Split-half analysis of BAVs. 18

Supplementary Tables

Table S1. Descriptions of behavioral measures from the IMAGEN project

Phenotype	Description of the behavioral scale	Description of sub-items	Source of behavioral scales	References for the scale
**ADHD**	ADHD symptom: Sum score of sub-symptoms; SDQ_Parent: Parent self-administered Strenghts and Difficulties Questionnaire using DAWBA interface	Restless (SDQ_Parent); Fidgety (SDQ_Parent); Easily Distracted (SDQ_Parent); Attentiveness (SDQ_Parent);Think before action (SDQ_Parent)	SDQ：Strength and DifficultiesStrengths and Difficulties Questionnaire	Goodman R. (1997). The Strengths and Difficulties Questionnaire: a research note. Journal of child psychology and psychiatry, and allied disciplines, 38(5), 581–586.
**Conduct Disorder**	Conduct Disorder symptom: Sum score of sub-symptoms; SDQ_Parent: Parent self-administered Strenghts and Difficulties Questionnaire using DAWBA interface	Fight or bully others (SDQ_Parent); Often lie (SDQ_Parent); Steal (SDQ_Parent)		
**Anxiety**	Anxiety symptom: Sum score of sub-symptoms; SDQ_Self: Child self-administered Strenghts and Difficulties Questionnaire using DAWBA interface	Many worries (SDQ_Self); Many fears (SDQ_Self); Anxious in new situations (SDQ_Self)		
**Depression**	Depression symptom: Sum score of sub-symptoms; SDQ_Self: Child self-administered Strenghts and Difficulties Questionnaire using DAWBA interface	Headache/stomach ache (SDQ_Self); Unhappy (SDQ_Self)		
**Alcohol−life**	ESPAD_Child: Child self-administered European School Survey Project on Alcohol and Drugs Questionnaire	Occasions IN WHOLE LIFETIME had any alcoholic beverage to drink (ESPAD)	ESPAD: European School Survey Project on Alcohol and Drugs	Hibell, B., Andersson, B., Bjarnason, T., Ahlström, S., Balakireva, O., Kokkevi, A., & Morgan, M. (2003). ESPAD
**Alcohol−year**		Occasions OVER THE LAST 12 MONTHS had any alcoholic beverage to drink (ESPAD)		
**Alcohol−month**		Occasions OVER THE LAST 30 DAYS had any alcoholic beverage to drink (ESPAD)		

**Smoking**		Smoke cigarettes ON A DAILY BASIS (ESPAD)		report. Alcohol and other drug use among students in, 35.
**Been Bully**	Being bullied by others	Been bullied at school; Been called mean names; Been excluded from their group of friends; Been hit, kicked, pushed or shoved around, or locked indoors by a peer; been bullied by a teacher; been bullied by a family member	Bully Questionnaire (BULLY; Olweus, 1996)	Olweus, D. (1996). Revised Olweus bully/victim questionnaire. Journal of Psychopathology and Behavioral Assessment.
**Bully**	Bullying others	took part in bullying another peer at school; called another peer mean names; kept a peer out of things on purpose; hit, kicked, pushed, shoved around, or locked a peer indoors; have bullied a teacher; have bullied a family member		
**PIQ**	Sum of Performance IQ score; The Wechsler Intelligence Scale for Children	Backward (DigitSpan); Forward (DigitSpan); Longest_backward (DigitSpan); Longest_forward (DigitSpan); Matrix Reasoning; Block Design	WISC-IV：The Wechsler Intelligence Scale for Children-Fourth Edition	O'Donnell, L. (2009). The Wechsler Intelligence Scale for Children—Fourth Edition.
**VIQ**	Sum of Verbal IQ score; The Wechsler Intelligence Scale for Children	Similarities; Vocabulary		
**Exploratory**	Temperament and Character Inventory – Revised	Exploratory excitability vs. stoic rigidity total (TCI)	TCI：Temperament and Character Inventory – Revised (TCI-R; Cloninger, et al. 1999)	Cloninger, C. R. (1999). The temperament and character inventory-revised. St Louis, MO: Center for Psychobiology of Personality, Washington University.
**Impulsiveness**		Impulsiveness vs. reflection total (TCI)		
**Extravagance**		Extravagance vs. reserve total (TCI)		
**Disorderliness**		Disorderliness vs. regimentation total (TCI)		
**Novelty Seeking**		Total Novelty Seeking score (TCI)		
**Externalizing Behaviours**	The sum of ADHD symptom score and Conduct Disorder symptom score; SDQ_Parent: Parent self-administered Strenghts and Difficulties Questionnaire using DAWBA interface	ADHD; Conduct Disorder	SDQ：Strength and DifficultiesStrengths and Difficulties Questionnaire	Goodman R. (1997). The Strengths and Difficulties Questionnaire: a research note. Journal of child psychology and psychiatry, and allied disciplines, 38(5), 581–586.
**Internalizing Behaviours**	The sum of Anxiety symptom score and Depression symptom score; SDQ_Self: Child self-administered Strenghts and Difficulties Questionnaire using DAWBA interface	Anxiety; Depression		

Table S2. Descriptions of behavioral measures from the HCP study

Phenotype	Description
**Anxious/Depression**	ASR Anxious/Depressed (scale I) Raw Score
**Attention Problem**	ASR Attention Problems (scale V) Raw Score
**Rule Breaking**	ASR Rule Breaking Behavior (scale VII) Raw Score
**Intrusive Score**	ASR Intrusive (scale VIII) Raw Score
**Internalizing Behaviors**	ASR Internalizing (scales I-III) Raw Score
**Externalising Behaviors**	ASR Externalizing (scales VI-VIII) Raw Score
**Drinks**	Total drinks in past 7 days. Asked on last day of HCP visit.
**Tobacco**	Total times used/smoked ANY TOBACCO in past 7 days. Asked on last day of HCP visit.
**Picture Vocabulary**	NIH Toolbox Picture Vocabulary Test: Unadjusted Scale Score

Table S3. Simulations of BAVs with different scenarios

Phenotype	Expected BAV	Observed BAV: mean (std)
noise(std=1)	0	mean = -0.001 (std = 0.01)
MID(std=1)+noise(std=4.36)	0.05	mean = 0.043 (std = 0.03)
MID(std=1)+noise(std=3)	0.1	mean = 0.102 (std = 0.06)
MID(std=1)+noise(std=2)	0.2	mean = 0.182 (std = 0.10)
MID(std=1)+noise(std=1)	0.5	mean = 0.477 (std = 0.25)

*MID stands for the normalised fMRI signals (*i.e.*, *std* = 1) from the monetary incentive delay task.

Table S4. Simulations of neuroimaging correlation with different scenarios

Phenotype 1	Phenotype 2	Expected neuroimaging correlation	Observed neuroimaging correlation: mean (std)
MID(std=1)+noise1(std=1)	SST(std=1)+noise2(std=1)	0	0.00 (0.27)
MID(std=5)+SST(std=1)+noise1(std=3)	MID(std=1)+ SST(std=5)+noise2(std=3)	0.38	0.39 (0.26)
MID(std=3)+SST(std=1)+noise1(std=3)	MID(std=1)+ SST(std=3)+noise2(std=3)	0.6	0.60 (0.30)
MID(std=1)+SST(std=1)+noise1(std=3)	MID(std=1)+ SST(std=1)+noise2(std=3)	1	0.91 (0.18)

*MID stands for the fMRI signals from the monetary incentive delay task; SST stands for the fMRI signals from the stop-signal task.

Table S5. Permutation P-values of the GMV-based BAVs from the IMAGEN

Phenotype	BAV	intercept	Quick P-value	Permutation P-value
ADHD	0.062	0.95	0.000	0.000
Conduct Disorder	0.022	1.04	0.011	0.014
Anxiety	-0.003	1.01	0.622	0.601
Depression	0.040	0.88	0.000	0.002
Alcohol−life	-0.002	1.04	0.506	0.480
Alcohol−year	-0.005	1.02	0.819	0.713
Alcohol−month	-0.003	1.04	0.597	0.519
Smoking	0.026	0.94	0.007	0.011
Been Bully	-0.001	0.99	0.449	0.438
Bully	0.004	1.05	0.192	0.197
PIQ	0.355	0.42	0.000	0.000
VIQ	0.196	0.61	0.000	0.000
Exploratory	-0.004	1.03	0.747	0.714
Impulsiveness	-0.004	1.02	0.745	0.734
Extravagance	0.001	1.04	0.299	0.309
Disorderliness	0.007	1.03	0.111	0.119
Novelty Seeking	-0.003	1.01	0.639	0.619
Externalizing Behaviors	0.069	0.92	0.000	0.000
Internalizing Behaviors	0.001	1.01	0.267	0.259

Table S6. BAVs of GMV from the IMAGEN project

Phenotype	BAV	Intercept	P-value	P_FDR
**ADHD**	0.062	0.95	0.000	0.000
**Conduct Disorder**	0.022	1.04	0.011	0.030
**Anxiety**	-0.003	1.01	0.622	0.759
**Depression**	0.040	0.88	0.000	0.000
**Alcohol-life**	-0.002	1.04	0.506	0.740
**Alcohol-year**	-0.005	1.02	0.819	0.819
**Alcohol-month**	-0.003	1.04	0.597	0.759
**Smoking**	0.026	0.94	0.007	0.022
**Been Bully**	-0.001	0.99	0.449	0.711
**Bully**	0.004	1.05	0.192	0.405
**PIQ**	0.355	0.42	0.000	0.000
**VIQ**	0.195	0.61	0.000	0.000
**Exploratory**	-0.004	1.03	0.747	0.789
**Impulsiveness**	-0.004	1.02	0.745	0.789
**Extravagance**	0.001	1.04	0.299	0.516
**Disorderliness**	0.006	1.03	0.111	0.264
**Novelty Seeking**	-0.003	1.01	0.639	0.759
**Externalizing Behaviors**	0.069	0.92	0.000	0.000
**Internalizing Behaviors**	0.001	1.01	0.267	0.507

Table S7. BAVs of GMV from the HCP study

Phenotype	BAV	Intercept	P-value	P_FDR
**Anxious/Depression**	0.011	0.99	0.333	0.363
**Attention Problem**	0.006	1.00	0.363	0.363
**Rule Breaking**	0.158	0.84	0.017	0.034
**Intrusive Score**	0.183	0.83	0.011	0.034
**Internalizing Behaviors**	0.022	0.98	0.261	0.326
**Externalizing Behaviors**	0.157	0.85	0.017	0.034
**Drinks**	0.135	0.92	0.025	0.042
**Tobacco**	0.059	0.98	0.119	0.170
**Picture Vocabulary**	0.318	0.71	0.000	0.000
**Cognition Total Score**	0.299	0.70	0.000	0.000

Table S8. BAVs of HCP Phenotypes with various VDI pattern

	VDI-HCP	VDI-IMAGEN-19	VDI-IMAGEN-14
**Phenotype-HCP**	**BAV**	**BAV**	**BAV**
Anxious/Depression	0.0107	0.0032	0.0019
Attention Problem	0.0058	0.0121	0.0066
Rule Breaking	0.1582	0.0316	0.0150
Intrusive Score	0.1834	0.0341	0.0143
Internalizing Behaviors	0.0221	0.0134	0.0042
Externalizing Behaviors	0.1565	0.0317	0.0132
Drinks	0.1347	0.0439	0.0117
Tobacco	0.0594	0.0155	0.0068
Picture Vocabulary	0.3177	0.0622	0.0350
Cognition Total Score	0.2986	0.0557	0.0292

Table S9. BAVs of MID-fMRI from the IMAGEN project

Phenotype	BAV	Intercept	P-value	P_FDR
**ADHD**	0.021	1.22	0.012	0.046
**Conduct Disorder**	0.046	1.02	0.000	0.000
**Anxiety**	0.001	0.94	0.284	0.385
**Depression**	0.007	1.10	0.110	0.224
**Alcohol−life**	-0.001	0.98	0.425	0.538
**Alcohol−year**	-0.003	1.05	0.645	0.766
**Alcohol−month**	0.006	1.00	0.118	0.224
**Smoking**	-0.004	1.16	0.834	0.885
**Been Bully**	-0.004	1.18	0.838	0.885
**Bully**	0.014	0.98	0.034	0.100
**PIQ**	0.109	0.74	0.000	0.000
**VIQ**	0.072	0.52	0.000	0.000
**Exploratory**	0.012	1.00	0.048	0.114
**Impulsiveness**	0.002	1.02	0.241	0.352
**Extravagance**	0.013	0.80	0.037	0.100
**Disorderliness**	-0.005	1.11	0.929	0.929
**Novelty Seeking**	0.003	0.78	0.208	0.329
**Externalizing Behaviors**	0.037	1.20	0.000	0.000
**Internalizing Behaviors**	0.005	1.05	0.136	0.235

Table S10. BAVs of SST-fMRI from the IMAGEN project

Phenotype	BAV	Intercept	P-value	P_FDR
**ADHD**	-0.001	1.38	0.439	0.491
**Conduct Disorder**	0.003	1.15	0.253	0.344
**Anxiety**	0.006	0.91	0.164	0.312
**Depression**	0.013	0.93	0.054	0.174
**Alcohol−life**	0.002	1.06	0.290	0.344
**Alcohol−year**	0.009	1.06	0.107	0.254
**Alcohol−month**	0.012	1.00	0.064	0.174
**Smoking**	0.013	0.98	0.055	0.174
**Been Bully**	0.007	0.93	0.137	0.289
**Bully**	0.013	1.04	0.059	0.174
**PIQ**	0.016	1.03	0.036	0.174
**VIQ**	0.013	1.23	0.059	0.174
**Exploratory**	0.005	1.03	0.207	0.344
**Impulsiveness**	0.003	1.06	0.288	0.344
**Extravagance**	-0.005	1.07	0.736	0.736
**Disorderliness**	-0.003	1.02	0.613	0.647
**Novelty Seeking**	0.004	1.04	0.232	0.344
**Externalizing Behaviors**	0.003	1.40	0.269	0.344
**Internalizing Behaviors**	0.013	0.91	0.055	0.174

Table S11. BAVs of EFT-fMRI from the IMAGEN project

Phenotype	BAV	Intercept	P-value	P_FDR
**ADHD**	0.005	1.27	0.148	0.404
**Conduct Disorder**	0.001	1.19	0.310	0.537
**Anxiety**	-0.003	1.07	0.566	0.710
**Depression**	0.008	0.89	0.112	0.404
**Alcohol−life**	0.005	0.93	0.149	0.404
**Alcohol−year**	0.007	0.93	0.124	0.404
**Alcohol−month**	-0.007	1.01	0.890	0.890
**Smoking**	-0.003	1.05	0.555	0.710
**Been Bully**	-0.002	1.06	0.515	0.710
**Bully**	0.010	1.09	0.080	0.404
**PIQ**	0.003	1.19	0.239	0.505
**VIQ**	0.027	0.94	0.010	0.190
**Exploratory**	0.001	1.26	0.311	0.537
**Impulsiveness**	-0.006	1.16	0.798	0.842
**Extravagance**	-0.005	1.05	0.737	0.824
**Disorderliness**	-0.003	1.07	0.576	0.710
**Novelty Seeking**	-0.003	1.07	0.598	0.710
**Externalizing Behaviors**	0.006	1.28	0.144	0.404
**Internalizing Behaviors**	0.003	0.98	0.215	0.505

Table S12. BAVs of Combined-MRI from the IMAGEN project

Phenotype	BAV	Intercept	P-value	P_FDR
ADHD	0.081	1.04	0.000	0.000
Conduct Disorder	0.092	1.01	0.000	0.000
Anxiety	0.010	0.99	0.231	0.298
Depression	0.055	0.97	0.005	0.014
Alcohol−life	-0.001	1.04	0.460	0.490
Alcohol−year	0.009	1.03	0.235	0.298
Alcohol−month	0.016	1.03	0.155	0.245
Smoking	0.045	1.00	0.012	0.029
Been Bully	-0.001	1.05	0.475	0.490
Bully	0.040	1.03	0.018	0.038
PIQ	0.308	1.29	0.000	0.000
VIQ	0.271	1.08	0.000	0.000
Exploratory	0.064	0.95	0.002	0.006
Impulsiveness	0.012	1.02	0.203	0.297
Extravagance	0.018	0.99	0.133	0.230
Disorderliness	-0.001	1.06	0.476	0.490
Novelty Seeking	-0.002	0.98	0.490	0.490
Externalizing Behaviors	0.112	1.02	0.000	0.000
Internalizing Behaviors	0.038	0.97	0.024	0.046

Table S13. Phenotypes in split-half analysis (age 14)

Phenotype	Description
**ADHD**	Restless (SDQ_Parent); Fidgety (SDQ_Parent); Easily Distracted (SDQ_Parent); Attentiveness (SDQ_Parent); Think before action (SDQ_Parent)
**Conduct Disorder**	Fight or bully others (SDQ_Parent); Often lie (SDQ_Parent); Steal (SDQ_Parent)
**Anxiety**	Many worries (SDQ_Self); Many fears (SDQ_Self); Anxious in new situations (SDQ_Self)
**Depression**	Headache/stomach ache (SDQ_Self); Unhappy (SDQ_Self)
**Hash**	Occasions OVER THE LAST 30 DAYS have you used marijuana (grass, pot) or hashish (hash, hash oil)
**Alcohol−life**	Occasions IN WHOLE LIFETIME had any alcoholic beverage to drink (ESPAD)
**Alcohol−year**	Occasions OVER THE LAST 12 MONTHS had any alcoholic beverage to drink (ESPAD)
**Alcohol−month**	Occasions OVER THE LAST 30 DAYS had any alcoholic beverage to drink (ESPAD)
**Smoking**	Smoke cigarettes ON A DAILY BASIS (ESPAD)
**Been Bully**	Been bullied at school; Been called mean names; Been excluded from their group of friends; Been hit, kicked, pushed or shoved around, or locked indoors by a peer; been bullied by a teacher; been bullied by a family member
**Bully**	took part in bullying another peer at school; called another peer mean names; kept a peer out of things on purpose; hit, kicked, pushed, shoved around, or locked a peer indoors; have bullied a teacher; have bullied a family member
**Exploratory**	Exploratory excitability vs. stoic rigidity total (TCI)
**PIQ14**	Backward (DigitSpan); Forward (DigitSpan); Longest_backward (DigitSpan); Longest_forward (DigitSpan); Matrix Reasoning; Block Design
**VIQ14**	Similarities; Vocabulary
**Extraversion**	Extraversion total (NEO)
**Agreeableness**	Agreeableness total (NEO)
**Conscientiousness**	Conscientiousness total (NEO)
**Impulsiveness**	Impulsiveness vs. reflection total (TCI)
**Extravagance**	Extravagance vs. reserve total (TCI)
**Disorderliness**	Disorderliness vs. regimentation total (TCI)
**NoveltySeeking**	Total Novelty Seeking score (TCI)
**Externalizing**	ADHD; Conduct Disorder
**Internalizing**	Anxiety; Depression

Table S14. R-values of behavior-based correlations

R-value	ADHD	Conduct Disorder	Depression	Smoking	Bully	Exploratory	PIQ	VIQ
**ADHD**		0.39	0.04	0.18	0.11	-0.02	-0.25	-0.22
**Conduct Disorder**			0.06	0.19	0.17	-0.03	-0.11	-0.12
**Depression**				0.05	0.07	0.00	-0.01	-0.01
**Smoking**					0.14	-0.01	-0.11	-0.11
**Bully**						0.07	-0.04	0.05
**Exploratory**							0.06	0.15
**PIQ**								0.45
**VIQ**								

Table S15. P-values of behavior-based correlations

P value	ADHD	Conduct Disorder	Depression	Smoking	Bully	Exploratory	PIQ	VIQ
**ADHD**		<0.001	0.151	<0.001	<0.001	0.530	<0.001	<0.001
**Conduct Disorder**			0.023	<0.001	<0.001	0.185	<0.001	<0.001
**Depression**				0.070	0.017	0.947	0.672	0.653
**Smoking**					<0.001	0.647	<0.001	<0.001
**Bully**						0.016	0.162	0.054
**Exploratory**							0.024	<0.001
**PIQ**								<0.001
**VIQ**								

Table S16. R_{brain}-values of neuroimaging correlation

r_{brain} value	ADHD	Conduct Disorder	Depression	Smoking	Bully	Exploratory	PIQ	VIQ
ADHD		1.00	0.99	1.03	0.64	-0.30	-0.81	-1.02
Conduct Disorder			1.24	0.91	0.79	-0.69	-0.84	-1.06
Depression				0.71	0.59	-1.08	-0.89	-1.02
Smoking					0.71	-0.28	-0.69	-0.70
Bully						-0.23	-0.58	-0.36
Exploratory							0.62	0.64
PIQ								0.94
VIQ								

Table S17. P-values of neuroimaging correlation

P_{perm} value	ADHD	Conduct Disorder	Depression	Smoking	Bully	Exploratory	PIQ	VIQ
ADHD		<0.001	<0.001	0.001	0.021	0.091	<0.001	<0.001
Conduct Disorder			<0.001	0.001	0.003	0.001	<0.001	<0.001
Depression				0.024	0.047	0.001	<0.001	<0.001
Smoking					0.038	0.212	<0.001	<0.001
Bully						0.325	0.001	0.019
Exploratory							<0.001	<0.001
PIQ								<0.001
VIQ								

Supplementary Figures

Flowchart of VDI pattern regression

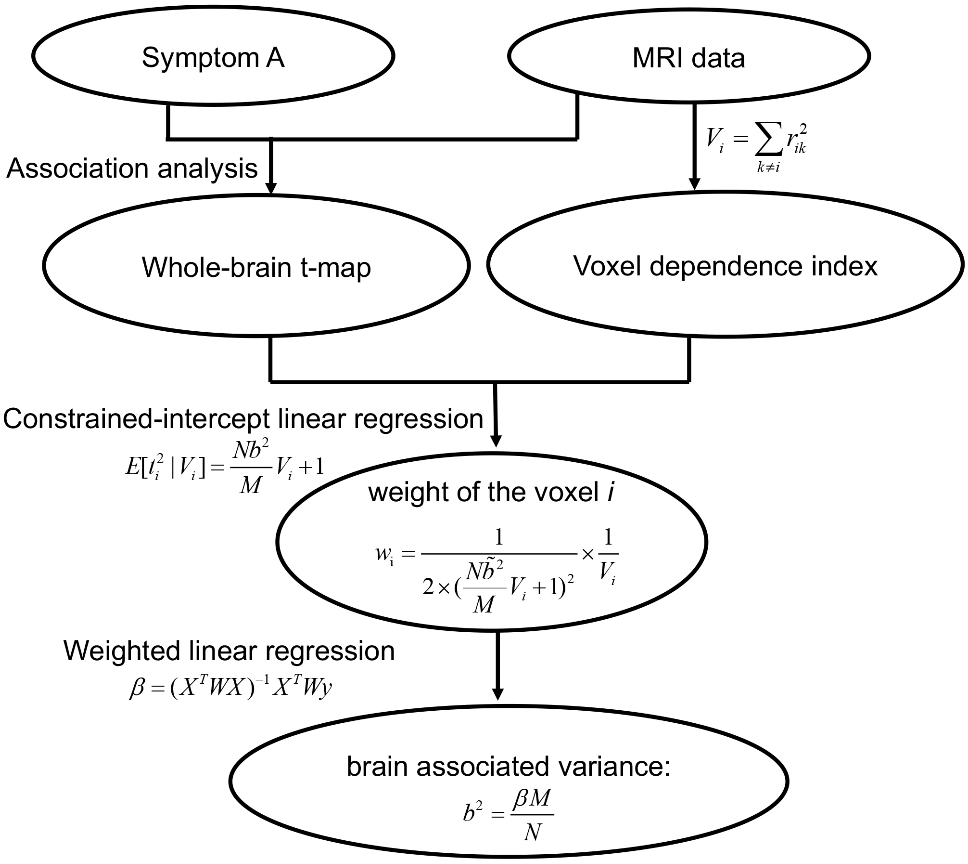

Figure S1. Flowchart of VDI pattern regression.

We first used the constrained-intercept linear regression, where the intercept was set to one, to estimate the conditional variance. We then applied the weighted linear regression to estimate the β, thus obtaining the value of BAV (*i.e.*, b^2). The N is the sample size, M is the number of voxels, V_i is the voxel dependence index (VDI) of voxel i. The b^2 is the BAV, analog to the genetic heritability.

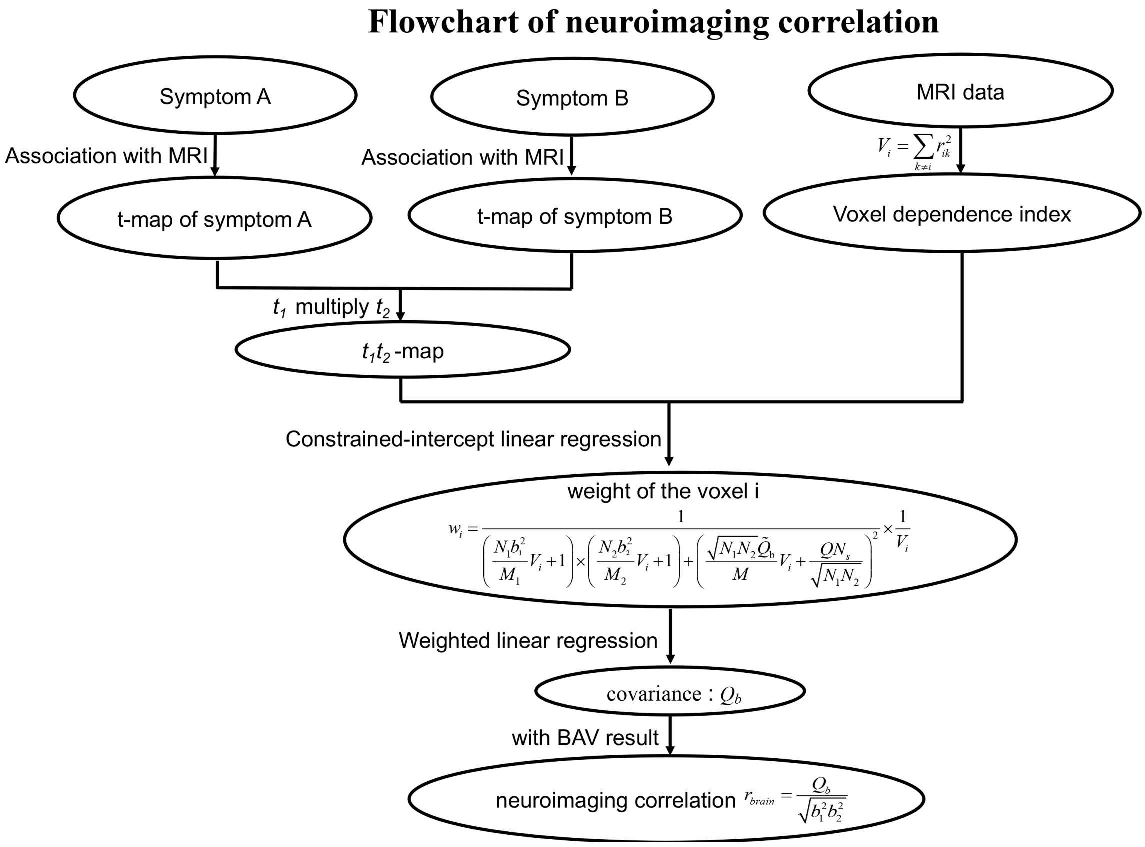

Figure S2. Flowchart of neuroimaging correlation.

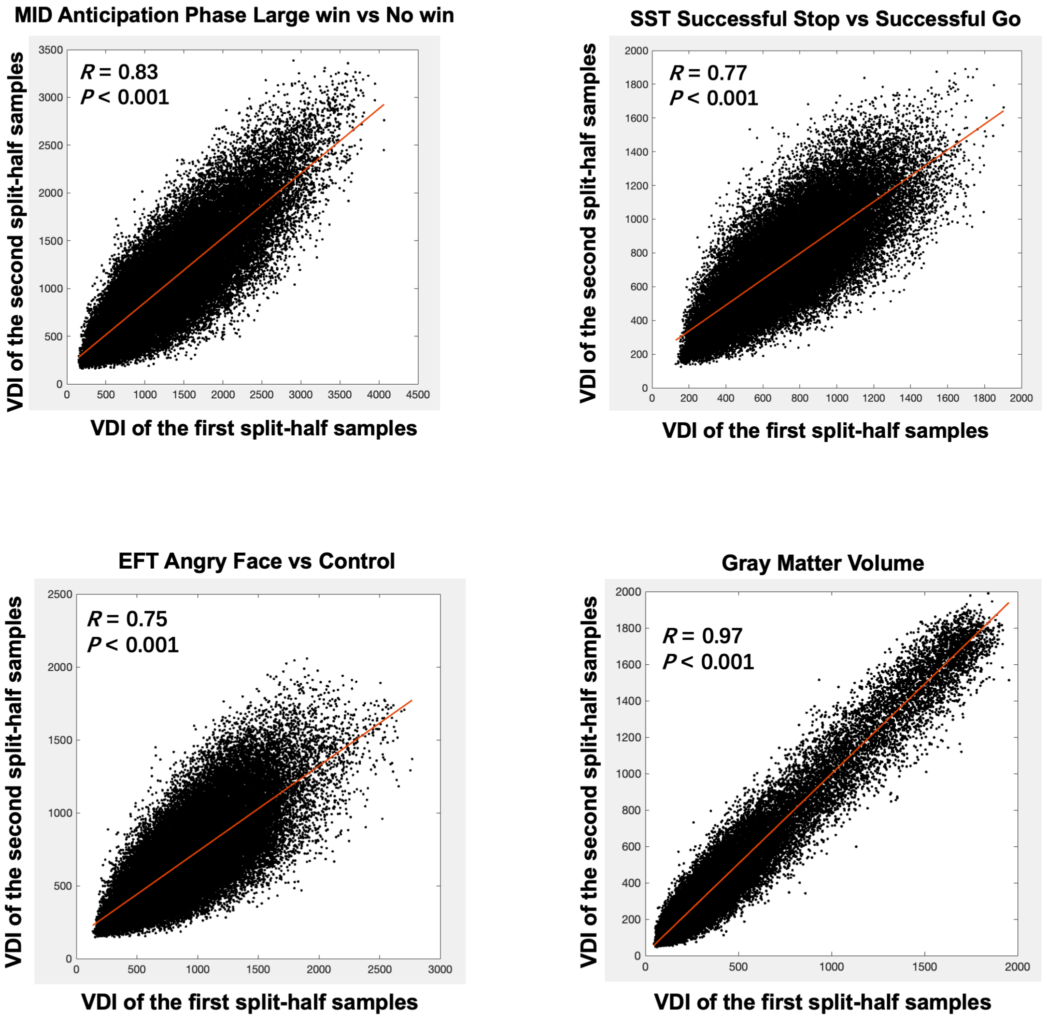

Figure S3. Split-half analysis of VDIs.

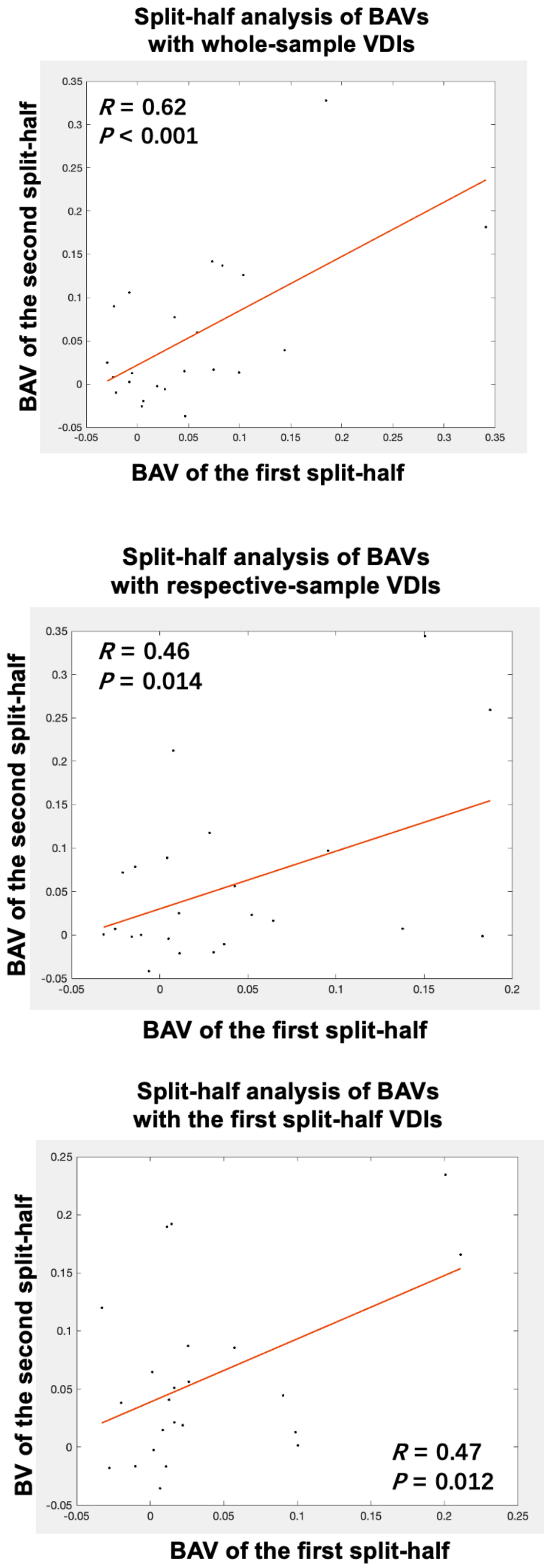

Figure S4. Split-half analysis of BAVs.
